# 

**Published:** 1887-08-27

**Authors:** 


					?Q ! &
PRICE ONE PENNY.
No. 48, Vol. II.
k. August 27th, 1887.
at the General Post Office
as a Newspaper.]
f Hi
u
Per Annam, 6s. 6d.,post free.
Monthly Parts, 6d.; post free, 7}d.
Ditto per Ann., 7s. 6d. post free.
Ili&Ui]
lUniiuimiiuiii
' an JnstituttoH, .jTanttlB, IParocftial journal o(
Tfeogpttafe, asplums. & all agencies for tf)e flare of tfie *>tcfe, Criticism & lietog.
mmmrvL

				

